# *In Vitro* Assessment of The Bioactive Compounds and Anticancer Potential of *Citrus medica* Leaf Extract

**DOI:** 10.21315/tlsr2023.34.3.11

**Published:** 2023-09-30

**Authors:** Mikkili Indira, Karlapudi Abraham Peele, Srirama Krupanidhi, Kodali Vidya Prabhakar, K.B.S. Vimala, P. Satya kavya, I. Sravya, T. C. Venkateswarulu

**Affiliations:** 1 Department of Biotechnology, Vignan’s Foundation for Science, Technology and Research, Vadlamudi-522213, Andhra Pradesh, India; 2 Department of Biotechnology, Vikrama Simhapuri University, Nellore-524004, Andhra Pradesh, India

**Keywords:** Antifungal, Antimicrobial, Antioxidant Activity, Flavonoids, Omega-3 Fatty Acids

## Abstract

*Citrus medica* is a horticultural crop grown in different parts of the world. The plant leaves have medicinal importance in traditional medicine for the treatment of various diseases. The leaves are an underutilised part of the plant, despite having various bioactive compounds with health benefits, with phytochemical analysis having revealed the presence of flavonoids, fatty acids, alkaloids, terpenoids, glycosides, carbohydrates and phytosterols. The biochemical constituents were identified using Fourier-transform infrared spectroscopy (FTIR) and gas chromatography–mass spectrometry (GC-MS), which confirmed the presence of terpenoids, alcohols, alkanes, phytosterols and fatty acids. Among these, methyl 8, 11, 14-heptadecatrienoate is a linolenic acid, and α-linolenic acid, trimethylsilyl ester and levulinic acid are the predominant compounds belonging to the omega-3 fatty acid group, which has known health benefits. Further, the antimicrobial activity of *C. medica* plant leaves were tested against certain food-borne pathogens and showed significant results. The minimum inhibitory concentrations ranged from 6.09 mg/mL to 390 mg/mL for bacterial organisms and 48.75 mg/mL to 390 mg/mL for fungal organisms. The antioxidant activity values were 300 μg/mL and 450 μg/mL by 2,2-diphenyl-1-picrylhydrazyl (DPPH) and 3-ethylbenzothiazoline-6-sulfonic acid (ABTS) assay, respectively. The methanolic extract from the *C. medica* leaves also showed anticancer activity against MCF7 breast cancer cell lines, with an IC50 value of material for developing a healthy processed food such as nutraceuticals and functional foods.

**Highlights**

- Antimicrobial activity of the *Citrus medica* leaves extract was tested against *Staphylococcus aureus and Candida albicans* was analysed through SEM. The extract is a rich source of bioactive compounds showed good antimicrobial, antioxidant and anticancer activity.
- The GC-MS analysis of leaf extract of *Citrus medica* revealed the presence of omega fatty acids which are considered to be as important as food supplements for boosting the immunity and also scavenging of free radicals.
- The active principles such as methyl 8, 11, 14-hepta decatrienoate which is a linolenic acid, α-linolenic acid trimethyl silyl ester and levulinic acid are the predominant compounds belongs to omega-3 fatty acids group with health benefits can be used in various food products that results in health benefits.

## INTRODUCTION

Increased consumption of processed foods and exposure to stress have both been linked to the increased prevalence of several chronic diseases (Shridhar *et al*. 2015; Khan *et al*. 2010). Further, the human body harbours microorganisms that can cause disease (Caputo *et al*. 2018). Risk of these diseases can be reduced by consumption of natural ingredients such as fruits, leaves and vegetables that contain biochemical constituents, mineral components, dietary fibers and vitamins (Rafiq *et al*. 2018; Kausar *et al*. 2019). Natural foods available in the nature are functional to some extent (Varzakas *et al*. 2016). However, some foods are now being examined for its health benefits to reduce the risk of chronic diseases (Alissa & Ferns 2012). The biochemical constituents have the ability to treat various ailments such as cardiovascular diseases, cancer, gastrointestinal disorders and physiological functions like lowering triglycerides and glucose control in blood (Asif 2011; Eilat-Adar *et al*. 2013; McClements & Xiao 2017; Eswaraiah *et al*. 2020).

Synthetic drugs used to treat these diseases can also affect healthy cells and cause adverse side effects (Karimi *et al*. 2015; Madigan & Karhu 2018). An alternative to these synthetic drugs is natural foods such as fruit and vegetables, which are natural sources of dietary fiber, vitamins and minerals (Bhaskarachary 2016). Compared with synthetic drugs, many fruits and vegetables deliver enhanced health benefits that exceed their nutritional value, termed functional foods, with biologically active substances such as antioxidants (Abuajah *et al*. 2015). Earlier studies also proved the relationship between functional ingredients of the food and health of wellbeing. The biochemical constituents of some functional foods positively impact health by reducing the risk of chronic diseases providing treatments for medical conditions such as cancer, cardiovascular disease and gastrointestinal disorders (Keservani *et al*. 2010; Das *et al*. 2012).

Developing functional foods which are supplements with added ingredients is currently a research priority in the developing world (Keservani *et al*. 2010). The natural food ingredients with antibacterial, antioxidant and anticancer properties have more importance in food processing industries (Cirmi *et al*. 2017; Adnan, Bibi, *et al*. 2014; Eswaraiah *et al*. 2019). Citrus plants have rich of bioactive molecules extracted from various parts of the plant used to treat a range of medical disorders (Lv *et al*. 2015; Chaudhari *et al*. 2016). *Citrus medica* plant extract has been used to treat arthritis, asthma, headaches, abdominal pain, intestinal parasites and psychological problems (Panara *et al*. 2012). *C. medica* leaves are rich in essential oils have huge demand in market and also the usage of these compounds increasing day by day. The *C. medica* plant is widely used in Chinese traditional medicine (Aliyah *et al*. 2017). This current study investigated the antimicrobial, antioxidant and anticancer properties of *C. medica* leaf extract. The antimicrobial property was tested against food borne pathogens, biochemical constituents by GC-MS analysis, antioxidant activity using DPPH and ABTS assay and anticancer activity against MCF7 breast cancer cell line.

## METHODS

### Collection of Plant Sample

The leaves of *Citrus medica (Citron-Dabbakaya)*, plant was collected from agricultural fields, Vijayawada, Krishna (Dt), Andhra Pradesh, India. Fresh leaves are collected during the winter season in the month of January 2018. The collected leaf samples were packed in plastic bags and transported to Biotechnology laboratory, Vignan’s Foundation for Science Technology and research for further work.

### Sample Preparation and Extraction of The Phytochemicals

Leaf samples were washed with sterile water to remove dust and foreign particles. The leaves were dried at 40°C in a hot-air oven for 3 days and then powdered. The Soxhlet extraction method was employed to extract the phytochemicals, using 100 g of powdered sample and 200 mL of methanol solvent at 12h intervals over three successive days at 65°C. For every hour, 3 cycles were run to extract the maximum number of compounds. After extraction, the methanol solvent was evaporated using a rotary evaporator under reduced pressure at 25°C for 1 h. The crude extract became a semi-solid mass and this was stored in Falcon tubes prior to further study (Adnan, Umer, *et al*. 2014).

### Phytochemical Screening of Citrus Leaves Extracts

The crude extract was tested for presence of phytochemicals such as alkaloids, glycosides, saponins, phenolic compounds, steroids, flavonoids, tannins, anthraquinones, amino acids, carbohydrates, terpenoids and phytosterols in the leaf sample (Ali *et al*. 2018).

### Fourier-Transform Infrared Spectroscopy

The functional groups of phytochemical compounds in the crude extract were identified using Fourier-transform infrared spectroscopy (FTIR) performed on a SHIMADZU FTIR–8400S. The crude extract 10 mg is mixed with 100 mg of KBr salt and compressed into thin pellet using mortar and pestle. The sample is loaded into spectroscope and the results were recorded at frequencies of 500 cm^−1^ to 4,000 cm^−1^ (Ashok Kumar & Ramaswamy 2014).

### Gas Chromatography–Mass Spectrometry

The phytochemical constituents in the crude extract were identified using gas chromatography–mass spectrometry (GC-MS) analysis, performed on Agilent^©^ 7890A-5975C equipment (Agilent Technologies, USA) using an HP 5 MS capillary column (30 m × 0.25 mm). The injection port conditions were: sample injected = 1 μL; carrier gas = helium with a flow rate of 1.2 mL/min; and temperature = 250°C. The GC column temperature was programmed initially at 80°C for 1 min, then the temperature was increased to 200°C at a rate of 15°C/min, further increased to 300°C at a rate of 5°C, and then maintained at 300°C for 5 min. The MS conditions were: temperature of the ion source = 230°C; ionisation energy = 70 eV; and a scan range of 50 amu–800 amu. The sample is prepared using methanol solvent and filtered. 50 μL of the sample is taken in 1.5 mL of autosampler and loaded into the injector port. The separated biochemical constituents were compared with the mass spectra in the NIST library (Rahman *et al*. 2019).

### Antimicrobial Activity Assay

#### Selection of indicator organisms and culture media

The antimicrobial activity of the leaf extract was performed by selecting the food borne pathogens such as bacteria and fungi. For antibacterial assay, indicator organisms are *Staphylococcus aureus* (MTCC 3103), *Escherichia coli* (MTCC 9537), *Enterobacter aerogenes* (MTCC 8558), *Pseudomonas aeruginosa* (MTCC 10306), *Klebsiella pneumoniae* (MTCC 10309), *Salmonella typhi* (MTCC 3224), *Shigella flexneri* (MTCC 9543), and *Bacillus subtilis* (MTCC 1305) were collected from Microbial Type Culture Collection (MTCC), Chandigarh, India. Nutrient agar medium and Muller Hinton agar medium was used for maintenance of cultures and antimicrobial activity, respectively. For antifungal assay, fungal cultures are *Fusarium oxysporum* (NCIM 1043), *Aspergillus niger* (NCIM 512), *Penicillium citrinum* (NCIM 766), *Trichoderma viridae* (NCIM 1051), and *Candida albicans* (NCIM 3471) were collected from National Collection of Industrial Microorganisms (NCIM), Pune, India.

### Agar Well Diffusion and Disk Diffusion Assay for Antimicrobial Activity

The selected indicator organisms were grown in a nutrient-broth medium and seeded for assay. Muller–Hinton agar plates (100 mm × 15 mm size) were prepared with 25 mL of medium and the inocula were seeded on the agar surface using the spread plate method. After lawn preparation, 4-mm-deep wells were created and loaded with 100 μL (1.6 × 10^8^ CFU/mL) of crude citrus-leaf extract (methanol) before the plates were incubated at 37°C for 24 h. The zone of inhibition was then measured and compared to both the positive control––ampicillin antibiotic––and the negative control––dimethyl sulfoxide (Sah *et al*. 2011). The selected fungal indicator organisms were grown in a Potato Dextrose Broth medium for 5 days to 7 days at 28°C–30°C. Muller–Hinton agar plates were prepared and seeded with inocula using a sterile swab dipped in culture suspension. The inoculated plates were then dried before applying the disks. Sterile disks were placed in 10 μL of crude leaf extract for 30 min and were then placed on the surface of the agar and incubated at 28°C for 5 days. The zone of inhibition was measured and compared to the positive control––fluconazole antibiotic––and the negative control––dimethyl sulfoxide. The procedure was repeated for three replicates and the mean values are calculated (Agarwal *et al*. 2015).

### Determination of Minimum Inhibitory Concentration

First, 10-mL measures of Mueller–Hinton broth medium were sterilised by autoclaving before being cooled and inoculated with 100 μL (1.6 × 10^8^ CFU/mL) of microbial cell suspension and 100 μL of plant extract (methanol) of known concentration. The concentrations of crude leaf extract used in this experiment were 0.76, 1.52, 3.05, 6.09, 12.18, 24.38, 48.75, 97.50, 195.00 and 390.00 mg/mL. The contents of the test tubes were mixed well and incubated at 37°C for 24 h. The procedure was repeated for three replicates and the mean values are calculated. The lowest concentration of crude leaf extract to inhibit the growth of microorganisms was calculated according to the method of Mummed *et al*. (2018), with some modifications.

### Antagonistic Activity Against Staphylococcus aureus and Candida albicans

The selected indicator organisms *S. aureus* and *C. albicans* were grown in nutrient broth and Sabouraud dextrose broth respectively at 35°C for 8 h (Li *et al*. 2019). After 8 h of incubation, the cells were separated by centrifugation at 5,000 rpm and suspended in phosphate-buffered saline (PBS) buffer with a pH of 7.4. The suspension (10^8^ CFU/mL) was mixed with leaf extract and incubated for 4 h. The cells were separated by centrifugation and washed with PBS buffer, then fixed with glutaraldehyde (2.5%) and stored at 4°C for 30 min. A 50% to 100% graded series of ethanol was used to dehydrate the sample for 15 min per concentration before the cells were imaged using an S-3700N scanning electron microscope (SEM, Hitachi, Hitachi City, Japan).

### Antioxidant Activity Assay

#### DPPH assay

The antioxidant potential of the *C. medica* leaf extract was measured by 2,2-diphenyl-1-picrylhydrazyl (DPPH) assay––a protocol suitable for the determination. Various concentrations of extract were prepared, in test tubes, ranging from 100 μg/mL to 1,000 μg/mL made up to a volume of 3 mL using 70% methanol. A 1-mL aliquot of 100-μM DPPH solution was added to each test tube, the mixture then shaken vigorously and incubated for 30 min. the absorbance was measured at 517 nm. Ascorbic acid was used as the control. All tests were performed in triplicate. The radical-scavenging activity was expressed as the inhibition percentage and was calculated using a standard formula. The antioxidant activity was also expressed as an IC_50_ value (Sonboli *et al*. 2010).

#### ABTS assay

The antioxidant activity of *C. medica* leaf extract was measured by 2,2’-azinobis (3-ethylbenzothiazoline-6-sulfonic acid (ABTS) assay. The reaction (1:1 v/v) was prepared via the oxidation of ABTS and potassium persulfate, with reaction allowed for 16 h under dark conditions. The mixture was then diluted with methanol until it achieved absorbance values of 1.0–1.5 at 734 nm. The leaf extract (0.1 mL) was mixed with 3.9 mL of ABTS solution and allowed to react for 2 h under dark conditions before the absorbance values were measured at 734 nm using a spectrophotometer. The results were expressed as a percentage of inhibition using the equation described for the DPPH method (Shah & Mehta 2018).

### Antiproliferative Activity of *Citrus medica*

A recognised MCF-7 breast cancer cell line was procured from the National Centre for Cell Science, Pune, India. Using RPMI-1640 medium, the cancer cells were sub-cultured in culture flasks and passaged every three days. The cells were then seeded in 24 well plates for 3-(4, 5-dimethylthiazolyl-2)-2, 5-diphenyltetrazolium bromide (MTT) assay. A series of crude leaf extract concentrations, ranging from 0 to 100 μg/mL, was prepared. First, the cells were trypsinzed and treated with trypan blue. Using a hemocytometer, the cells were counted and seeded at a density of 5.0 × 10^3^ cells/well in 96 well plates, then incubated at 37°C overnight. The medium was then replaced with fresh medium and 100 μL of different concentrations of crude leaf extract were added. The cells were incubated for 48 h, after which fresh medium and MTT solution (0.5 mg/mL) were added to each well. The cells were then incubated for a further 3 h, after which the absorbance values were measured at 570 nm using a microplate reader. The percentage growth inhibition and corresponding IC_50_ values were generated from the dose-response curve using Origin software (Stockert *et al*. 2018).

## RESULTS

### Phytochemical Screening

During the present study, the phytochemical constituents were identified by performing various tests and found the presence of alkaloids, glycosides, flavonoids, steroids, terpenoids, carbohydrates and phenolic compounds (Table 1). There is an absence of saponins, anthraquinones and tannins.

### FTIR and GC–MS

**Figure 1:**
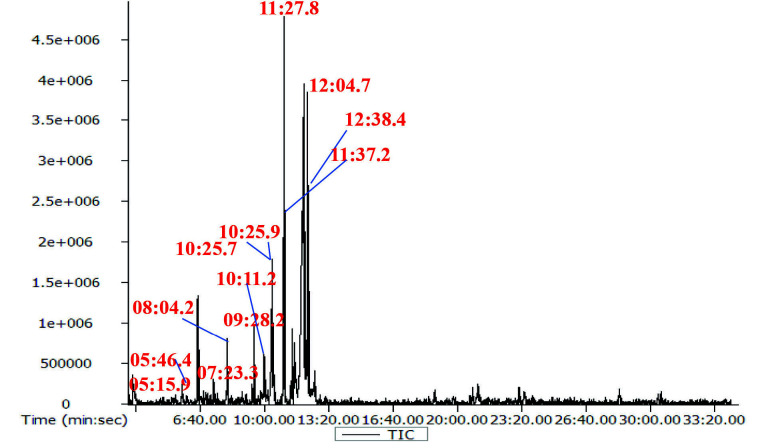
GC–MS analysis of *C. medica* leaves.

The FTIR analysis revealed the presence of various functional groups and biochemical classes of compounds in the leaf extract (Table 2). Fig. 1 presents the GC–MS spectra, showing various peaks representing different bioactive compounds.

**Figure 2:**
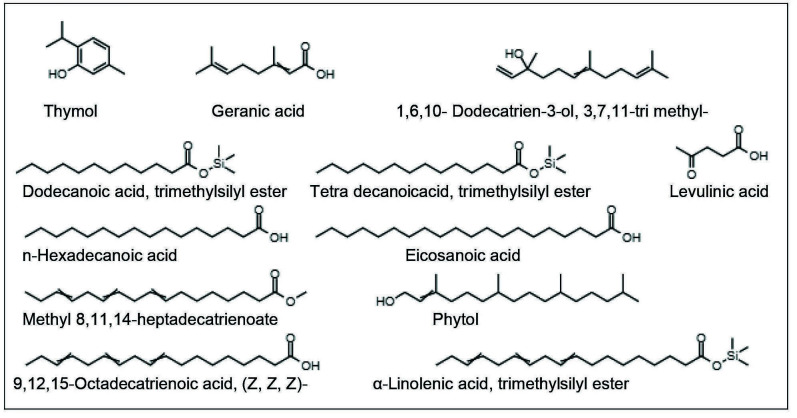
Structures of compounds identified by GC-MS analysis.

The compounds were identified from the methanolic extract of the *C. medica* leaves, as indicated in Table 3 and Fig. 2.

### Antimicrobial Activity

**Figure 3:**
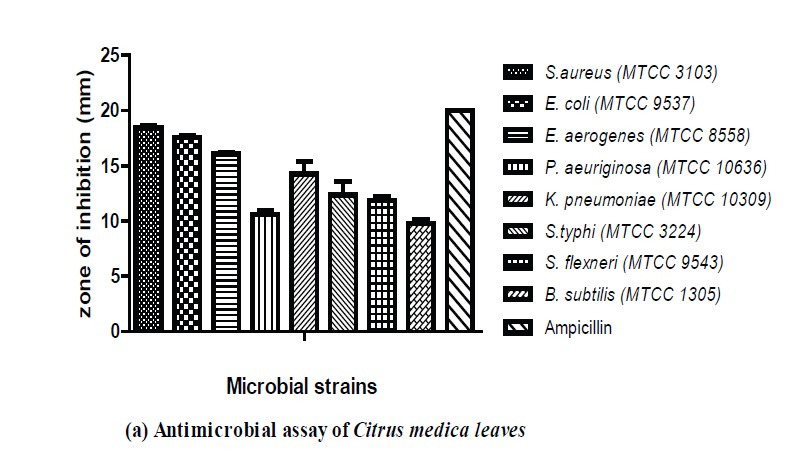

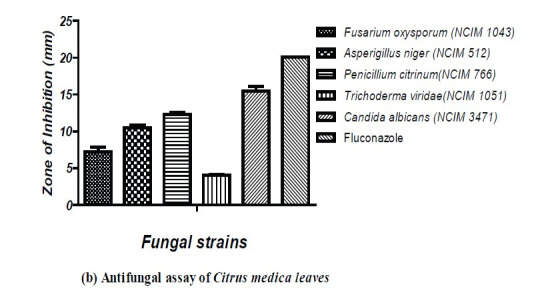
(a) Antimicrobial activity of *C. medica* leaf extract; (b) Antifungal activity of *C. medica* leaf extract.

The antimicrobial activity of the methanolic extract was tested against selected indicator organisms, with the results provided in Figs. 3a and 3b. These show that the highest antimicrobial activity was observed for the strain *Staphylococcus aureus* (18.46 ± 0.04 mm), followed by *Escherichia coli* (17.53 ± 0.06 mm), *Enterobacter aerogenes* (16.06 ± 0.06 mm), and *Klebsiella pneumonia* (14.26 ± 1.6 mm). Moderate inhibition was observed for the strains *Salmonella typhi* (12.36 ± 2.0 mm), *Pseudomonas aeruginosa* (10.6 ± 0.9 mm), *Shigella flexneri* (11.9 ± 0.06 mm), and *Bacillus subtilis* (9.8 ± 0.06 mm). The results were compared with the ampicillin control. From the antifungal assay, it was found that *Candida albicans* (15.46 ± 1.2 mm) is very sensitive to the *C. medica* extract, followed by *Penicillium citrinum* (12.3 ± 0.09 mm), *Aspergillus niger* (10.46 ± 1.0 mm), and *Fusarium oxysporum* (7.26 ± 1.2 mm). The strain *Trichoderma viride* (4.06 ± 0.04 mm) was less sensitive to the extract compared with the standard drug fluconazole. All the results are written as mean of three individual observations ± SD.

### Minimum Inhibitory Concentration

The minimum inhibitory concentration (MIC) of *C. medica* leaf extract was determined and the values found to range from 6.09 mg/mL to 390 mg/mL. High antibacterial activity was observed for the MIC value of 6.09 mg/mL against *E. coli*, *S. aureus* and *E…aerogenes*. For the fungal cultures, considerable antibacterial activity was observed at a MIC value of 48.75 mg/mL against *C. albicans* (Table 4).

### Antagonistic Activity Against *S. aureus* and *C. albicans*

**Figure 4:**
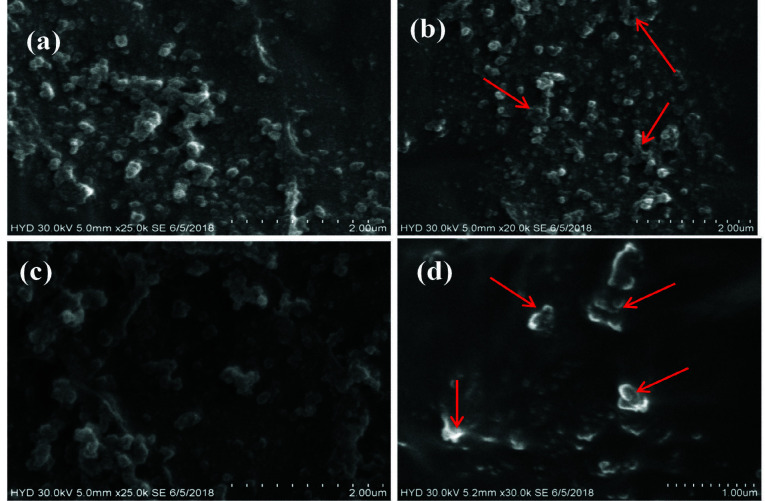
Antagonistic activity of Citrus medica leaf extract on *S. aureus* and *C. albicans*. (a) Untreated cells of *S. aureus;* (b) Treated cells of *S. aureus;* (c) Untreated cells of *C. albicans;* and (d) Treated cells of *C. albicans*.

Antagonistic activity was evaluated against the food-borne pathogens *S. aureus* and *C. albicans*. The effect of *C. medica* leaf extract on the cell morphology of *S. aureus* and *C. albicans* was investigated using a scanning electron microscope (at 50,000× magnification) by comparing the morphological features of both treated and untreated cells. Untreated *S. aureus* cells presented as aggregations of rounded cells (Fig. 4a), whereas the treated cells varied in size and shape and demonstrated some cell shrinkage (Fig. 4b). The untreated cells of *C. albicans* were characterised by their regular round or oval shapes Fig. 4c), whereas the treated cells were characterised by ruptured hyphae and cell membranes (Fig. 4d).

### Antioxidant Activity Assay

The methanolic extract of *C. medica* demonstrated strong antioxidant activity by reducing the formation of DPPH radicals by 50% at an IC_50_ value of 300 μg. The mean IC_50_ value of ascorbic acid was found to be 50 μg. In the case of the ABTS assay, the free-radical ABTS inhibition by methanolic extract was found to be 450 μg (Table 5).

**Figure 5:**
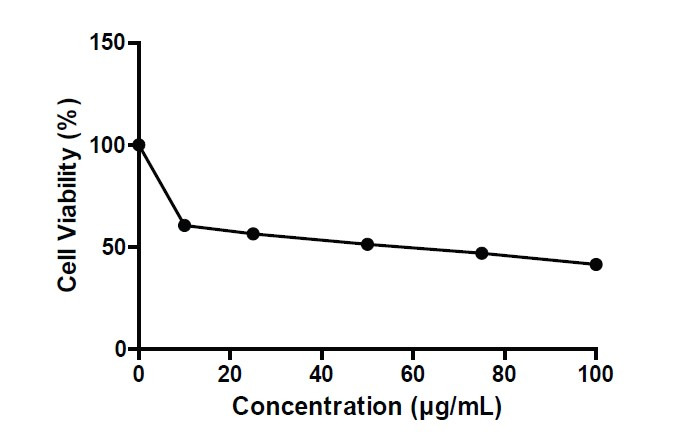
Cytotoxic activity of methanolic extract of *C. medica* plant leaves against MCF7 cell lines.

The methanolic extract of *C. medica* plant leaves was evaluated for its antiproliferative activity against the MCF7 breast cancer cell line by MTT assay. An inhibition of viable MCF7 cells counted after treatment with the extract was observed. The results revealed that the cell line viability was decreased gradually with an increase in sample concentration. The maximum reduction in cells was found at a concentration of 100 μg/mL, with the viability being 41.473%. The IC_50_ value was 60.044 μg/mL at 48 h on the MCF7 cell line (Fig. 5).

## DISCUSSION

Citrus leaves are rich in various biochemical compounds particularly phenolic compounds and flavonoids (Khettal *et al*. 2017). The phytochemicals screened in the citrus leaf extract are alkaloids, flavonoids, carbohydrates, glycosides, terpenoids and phenolic compounds. In recent study by Chhikara *et al*. (2018) also reported the presence of alkaloids, glycosides, flavonoids and steroids in the leaves of *C. medica*. In another study by Patil (2017) reported the presence of alkaloids, steroids, glycosides and phenols in the citrus plant. The FTIR analysis study revealed the presence of functional groups such as halogen compounds (C-I, C-Br and C-Cl stretching), primary and secondary alcohols (C=O stretch), aromatic amine (C-N stretch), aromatic compounds (C=C stretch, C-H bending), amines (N-H bending and N-H stretch), thiocyanate (S-C=N stretch), isothiocyanate (N=C=S stretching), isocyanate (N=C=O stretch), nitrile compounds (C=N stretch) and carboxylic acids (O-H stretching) represented in Table 2. In previous study, Onyeyirichi *et al*. (2014) reported that the Citrus medica Limonium leaf essential oil consists of alcohols, amines, alkyl halides and alkanes.

Further, the GC-MS analysis showed 12 major peaks (Fig. 1) and the details of compounds are shown in Table 3. The major peak compounds are methyl 8,11,14-heptadecatrienoate, 9,12,15-octadecatrienoic acid, (Z, Z, Z)-, α-linolenic acid trimethylsilyl ester and phytol. The compounds with biological activity identified are thymol (antioxidant), geranic acid (pheromone and antiseptic material), dodecanoic acid, trimethylsilyl ester (surfactant) n-hexa decanoic acid (anti-inflammatory) (Nagoor *et al*. 2017; Greene *et al*. 2019; Qiao & Qiao 2013; Aparna *et al*. 2012). The compound 1, 6, 10-dodecatrien-3-ol, 3, 7, 11-tri methylis also known as nerolidol which is a flavour enhancer and levulinic acid is an additive (Chan *et al*. 2016; Zhou *et al*. 2020). The other compound is eicosanoic acid (Arachidonic acid), which is a saturated fatty acid (omega-6-fatty acid) and starting material for synthesis of prostaglandins (Powell & Rokach 2015). Apart from these, the abundant compounds with application in food industry are phytol, methyl 8, 11, 14-hepta decatrienoate, 9,12,15-octadeca trienoic acid, (Z, Z, Z)- and α-linolenic acid, trimethylsilyl ester. Phytol is a precursor for synthesis of vitamin E and Vitamin K (Byju *et al*. 2017). Methyl 8, 11, 14-hepta decatrienoate and 9,12,15-Octadeca trienoic acid, (Z, Z, Z)- both are having same molecular formula (C_18_H_3_0O_2_) called as linolenic acid. The linolenic acid and α-linolenic acid, trimethyl silyl ester are the essential omega 3- fatty acids group highly concentrated in plant oils (Blondeau *et al*. 2015). The linolenic acid and alpha linolenic acids have been reported to inhibit the synthesis of prostaglandins leads to reduced inflammation in various chronic disorders (Glick & Fischer 2013). In recent study, Aliyah et al. (2017) found the presence of citral, limonene, linalool, citronella, geranyl acetate, 2-hexadecen-1-ol,3,7,11,15-tetramethyl- [R[R*R*-(E]] (cas) phytol in leaf essential oils. The variation of the compounds present in the plant leaves is due to changes in geographical, physiological and environmental factors. From FTIR analysis and GC–MS analysis it was found that the *C. medica* leaves consist of alcohols, alkanes, terpenoids, phytosterols and fatty acids. In another study reported by Lv *et al*. (2015) found that the citrus fruits also contain the ingredients such as flavonoids, phenolic compounds, terpenoids, polysaccharides and amino acids. The compounds present in the *C. medica* leaves extract are similar to the biochemical constituents present in the fruits, which are beneficial to human health.

The antimicrobial activity was studied against the selected food borne pathogens. In support of the findings of this study, the inhibition of microbial growth by *C. medica* extract has previously been demonstrated for *S. aureus* (Aliyah *et al*. 2017; Sah *et al*. 2011; Li *et al*. 2019), *E. coli* (Li *et al*. 2019), *B. subtilis* (Li *et al*. 2019) and *C. albicans* (Aliyah *et al*. 2017). However, Sah *et al*. (2011) also failed to detect any inhibition of growth of *P. aeruginosa*, *K. pneumoniae*, *E. coli*, *P. vulgaris*, *A. niger*, *A. flavus* and *C. albicans* when exposed to *C. medica* extract. Further, previous studies have also found that *C. medica* extract inhibited the growth of some microorganisms not tested during this study, including *E. faecalis* (Sah *et al*. 2011), *M. luteus* (Li *et al*. 2019) and *P. acne* (Aliyah *et al*. 2017). In addition to this, the antagonistic activity was evaluated by selecting the *S. aureus* and *C. albicans* through Scanning electron microscopy and found changes in morphology, ruptured cell membranes and leakage of cellular constituents were observed. Similar type of study by Li *et al*. (2019) was reported for *E. coli* and *S. aureus* treated with citron essential oil.

In our body, the free radicals are produced and reacts with tissue causes oxidative damage. Our body has complex system of antioxidant defense mechanism of enzymes such as catalase, glutathione peroxidase and superoxide dismutase. However, under certain conditions, there is an imbalance occurs due to excessive production of free radicals results in oxidative stress (Khettal *et al*. 2017). Antioxidants are the chemicals that inhibits the oxidation and counteract the oxidative damage (Shah & Mehta 2018). Many studies illustrate the importance of natural antioxidants usage in the food processing industry and medical fields. The natural antioxidants have protective role against the reactive oxygen species due to the presence of bioactive compounds. In the present study, the methanolic extract was examined for *in-vitro* antioxidant activity through DPPH assay and ABTS assay. For DPPH assay and ABTS assay the IC_50_ values are found to be 300 μg and 450 μg, respectively. There are no reports available related to anticancer activity of *C. medica* leaves. In this study, the anticancer activity was evaluated against MCF-7 breast cancer cell line. In previous study by Entezari *et al*. (2009) reported the anti-mutagenicity and anticancer effect of citrus fruit juice.

## CONCLUSION

A GC–MS analysis of *C. medica* leaves confirmed the presence of various bioactive compounds with biological activity and food applications. The *C. medica* leaf extract showed good antioxidant, anticancer and antimicrobial activity against food-borne pathogens. The abundant compounds present in the extract were linolenic acid and α-linolenic acid trimethylsilyl ester compounds belonging to the omega-3 fatty acid group. Omega-3 fatty acids have a beneficial role in cancer-related complications, as well as having antioxidant properties. Omega-6 fatty acid––eicosanoic acid plays a beneficial role in acting against inflammation and cardiovascular disease. This study indicates the potential for the development of *C. medica* extract as a natural functional food supplement for reducing the risk of chronic diseases.

## Figures and Tables

**Table 1:** Phytochemical analysis of methanolic extract of *C. medica*.

Sample no.	Phytochemical	Inference
1	Alkaloids	+
2	Glycosides	+
3	Saponins	−
4	Steroids	+
5	Flavonoids	+
6	Tannins	−
7	Anthraquinones	−
8	Carbohydrates	+
9	Phenolics	+
10	Terpenoids	+
11	Phytosterols	+

*Notes:* + indicates Present; – indicates Absent

**Table 2:** FTIR analysis of phytoconstituents of methanol leaf extract of *C. medica*.

Wave number (cm^−1^)	Possible bonds	Intensity
503.44, 549.73, 569.02, 582.52,	C-I stretching and C-Br stretching	Strong
669.32, 704.04,	C-Cl stretching, C=C bending	Strong
1026.16, 1141.90,	C=O stretch	Strong
1271.13, 1315.50, 1336.71	C-N stretch	Medium-weak
1408.08, 1433.16, 1518.03, 1579.75	C=C stretch	Medium-weak
1639.55	N-H bending	Medium
1872.94, 1911.52,1988.68	C-H bending	Weak
2065.83, 2090.91,	N=C=S stretching	Strong
2162.27	S-C=N stretch	Strong
2224.00	C=N stretch	Weak
2266.43	N=C=O stretch	Strong
2526.83,2596.27,2708.15, 2854.74, 2916.47, 3001.34	O-H stretching	Strong
3419.9, 3444.98	N-H stretch	Medium

**Table 3:** GC-MS analysis for leaf extract of *C. medica*.

Sample no.	Name	Formula	RT	(RI)	(SI)	NIST-RI	CAS number	Integrated peak area	Exact mass
1	Thymol	C_10_H_14_O	05:15.9	1195.3	929	1266	89-83-8	170323	150.1045
2	Geranic acid	C_10_H_16_O_2_	05:46.4	1253.3	949	1342	459-80-3	3879701	168.115
3	1,6,10-dodecatrien-3-ol,3,7,11-tri methyl-	C_15_H_26_O	07:23.3	1445.8	960	1545	40716-66-3	5746258	222.1984
4	Dodecanoic acid, trimethylsilylester	C_15_H_32_O_2_Si	08:04.2	1532.7	839	1590	55520-95-1	1234539	272.2172
5	Tetradecanoic acid, trimethylsilylester	C_17_H_36_O_2_Si	09:28.2	1722.5	948	1840	18603-17-3	11499156	300.2485
6	Levulinic acid	C_5_H_8_O_3_	10:11.2	1817.6	999	1823	123-76-2	3355465	116.0473
7	n-hexadecanoic acid	C_16_H_32_O_2_	10:25.7	1847.3	865	1964	57-10-3	72544533	256.2402
8	Eicosanoic acid	C_20_H_40_O_2_	10:25.9	1847.7	828	2359	506-30-9	72437943	312.3028
9	Methyl 8,11,14-heptadecatrienoate	C_18_H_30_O_2_	11:27.8	1966.9	865	2002	155273-05-5	17068620	278.2246
10	Phytol	C_20_H_40_O	11:37.2	1984.2	888	2045	150-86-7	4333265	296.3079
11	9,12,15-octadecatrienoic acid, (Z, Z, Z)-	C_18_H_30_O_2_	12:04.7	2031.3	946	2073	463-40-1	296511194	278.2246
12	α-linolenic acid, trimethylsilyl ester	C_21_H_38_O_2_Si	12:38.4	2087.1	768	2191	97844-13-8	15316759	350.2641

*Notes*: RT = Retention time; RI = Retention index; SI = Similarity index

**Table 4:** MIC values of *C. medica* leaf extract versus selected indicator organisms.

Sample no.	Indicator organism	MIC concentration (mg/mL)
1	*Staphylococcus aureus*	6.09 ± 0.00
2	*Pseudomonas aeruginosa*	195 ± 0.00
3	*Enterobacter aerogenes*	6.09 ± 0.00
4	*Kl*e*bsiella pneumoniae*	12.18 ± 0.00
5	*Escherichia coli*	6.09 ± 0.00
6	*Salmonella typhi*	48.75 ± 0.00
7	*Shigella flexneri*	48.75 ± 0.00
8	*Bacillus subtilis*	390 ± 0.00
9	*Fusarium oxysporum*	390 ± 0.00
6	*Aspergillus niger*	195 ± 0.00
7	*Penicillium citrinum*	97.5 ± 0.00
8	*Trichoderma viride*	390 ± 0.00
9	*Candida albicans*	48.75 ± 0.00

**Table 5:** IC_50_ values for *C. medica* leaf extract (antioxidant assay).

Sample no.	Extract type	IC_50_ value(μg)	

		DPPH radical assay	ABTS radical assay
1	Methanolic extract	300	450
2	Ascorbic acid	50	60
